# Association of long-term exposure to PM_2.5_ with hypertension and diabetes among the middle-aged and elderly people in Chinese mainland: a spatial study

**DOI:** 10.1186/s12889-022-12984-6

**Published:** 2022-03-22

**Authors:** Zirong Ye, Xueru Li, Yaofeng Han, Yafei Wu, Ya Fang

**Affiliations:** 1 State Key Laboratory of Molecular Vaccine and Molecular Diagnostics, School of Public Health, Xiamen University, Xiamen, China; 2 Key Laboratory of Health Technology Assessment of Fujian Province, School of Public Health, Xiamen University, Xiamen, China; 3 National Institute for Data Science in Health and Medicine, Xiamen University, Xiamen, China

**Keywords:** PM_2.5_, Hypertension, Diabetes, Spatial study

## Abstract

**Background:**

Epidemiological evidence has shown an association between long-term exposure to fine particulate matter (PM_2.5_) and hypertension and diabetes, but few studies have considered the spatial properties of the samples. This study aimed to investigate the long-term effect of PM_2.5_ exposure on hypertension and diabetes among middle-aged and elderly people in China based on a spatial study.

**Methods:**

We conducted a national cross-sectional study of the most recently launched wave 4 2018 data of the China Health and Retirement Longitudinal Study (CHARLS) to calculate the prevalence of hypertension and diabetes. The exposure data of annual average PM_2.5_ concentrations were estimated combined with satellite observations, chemical transport modeling, and ground-based monitoring. A shared component model (SCM) was used to explore the association of PM_2.5_ with hypertension and diabetes, in which these two diseases borrowed information on spatial variations from each other. Then, we evaluated the effect variations in PM_2.5_ in different periods and smoking status on changes in outcomes.

**Results:**

The prevalence of hypertension and diabetes was 44.27% and 18.44%, respectively, among 19,529 participants. The annual average PM_2.5_ concentration in 31 provinces ranged from 4.4 μg/m^3^ to 51.3 μg/m^3^ with an average of 27.86 μg/m^3^ in 2018. Spatial auto-correlations of the prevalence of hypertension and diabetes and PM_2.5_ concentrations were seen (Moran’s *I* = 0.336, *p* = 0.01; Moran’s *I* = 0.288, *p* = 0.03; Moran’s *I* = 0.490, *p* = 0.01). An interquartile range (IQR: 16.2 μg/m^3^) increase in PM_2.5_ concentrations was significantly associated with a higher prevalence of hypertension and diabetes with odds ratios (ORs) of 1.070 [95% credible interval (95% CrI): 1.034, 1.108] and 1.149 (95% CrI: 1.100, 1.200), respectively. Notably, the effect of PM_2.5_ on both hypertension and diabetes was relatively stronger among non-smokers than smokers.

**Conclusion:**

Our nationwide study demonstrated that long-term exposure to PM_2.5_ might increase the risk of hypertension and diabetes, and could provide guidance to public policymakers to prevent and control hypertension and diabetes according to the spatial distribution patterns of the above effects in China.

**Supplementary Information:**

The online version contains supplementary material available at 10.1186/s12889-022-12984-6.

## Background

Hypertension and diabetes are common chronic diseases worldwide. Approximately one-third of the adults aged 20 years or older in the world were reported to have hypertension [1]. The global prevalence of diabetes was expected to reach 10.2% (578 million) by 2020 and 10.9% (700 million) by 2045 [2]. As a developing country, the prevalence of hypertension and diabetes in China has remained at a relatively high level. According to the China Hypertension Survey Study, the prevalence of hypertension was about 23.2% in 2015 [3]. And the latest epidemiological study showed that approximately 11% of the population in China had diabetes [4]. The prevalence of hypertension and diabetes should continue to rise with the rapid aging of the Chinese population [5]. To improve the efficiency of prevention, determining the risk factors of hypertension and diabetes over a geographic area are crucial. It is worth noting that hypertension and diabetes share common risk factors, such as individual behavior, family history, genetic factors, and lifestyle [6–8]. Due to some shared pathogenic mechanisms, these two diseases coexist frequently and have similar spatial distribution patterns.

A few epidemiological studies have shown that both short-term and long-term exposure to PM_2.5_ was significantly associated with the prevalence of hypertension and diabetes. Two study by Liu et al. on the effect of long-term exposure to PM_2.5_ suggested that increases in PM_2.5_ were associated with a higher prevalence of hypertension and diabetes [9, 10]. Two cohort studies by Gu et al. also found similar positive associations in China [11, 12], whereas other studies suggested that there was no significant association between long-term exposure to PM_2.5_ and hypertension and diabetes [13, 14]. Variations in population characteristics, spatial regions, pollutant sources and composition, and exposure measurements may lead to inconsistencies in the results. Since the pathogenesis of hypertension and diabetes is complex, utilizing the location may serve as a useful surrogate for investigating the mixture of the above confounding factors that may underlie any spatial variations in disease risk. Applying spatial information offers further control of the confounding factors, which, in turn, can reveal the real association of PM_2.5_ with hypertension and diabetes.

With the development of the Markov chain Monte Carlo (MCMC) method, multiple methods have been derived from the Bayesian hierarchical model (BHM), including the shared component model (SCM) which is an advancement of the BHM. The SCM was first proposed by Knorr and Best [15] for the joint spatial analysis of two diseases and extended to multiple diseases by Held et al. [16]. The basic assumption of the SCM is that many diseases may depend upon each other and share common risk factors. Thus, the key idea of the model is to separate the underlying risk for each disease into a shared component, common to both diseases, and a disease-specific component. The shared component is further divided into two parts, which can be interpreted as surrogates for unobserved covariates that display spatial structures and non-spatial structures. Similarly, the disease-specific component represents the spatially varying risk factors and non-spatially varying risk factors that are specific to the respective disease. Therefore, when diseases with similar spatial distribution characteristics and risk factors are modeled jointly, one disease is used as a surrogate for the unobserved risk factors of the other disease. Different from traditional statistical methods, the SCM modeling procedure utilizes dependency, not only among diseases but also among spatially varying variables. This model can better control the unobserved confounding factors and describe the epidemiologic features of the risk factors associated with a certain disease [17]. Recently, the SCM has been applied to analyze gender variations in disease risk, the effects of PM_2.5_ on hypertension, estimate the relative risk of multiple cancers, and recognize spatial patterns [18–20].

Considering that hypertension and diabetes are two highly prevalent chronic diseases, both have similar spatial distribution patterns and risk factors. Therefore, this study was proposed to explore the association between long-term PM_2.5_ exposure and the prevalence of hypertension and diabetes simultaneously by applying SCM from a spatial perspective. Furthermore, some studies suggested that the impact of PM_2.5_ on humans varied among people with different smoking statuses [21]. Thus, we explored the effect of smoking status on association changes. Our study was based on the nationally representative China Health and Retirement Longitudinal Study (CHARLS) survey, which provided a high-quality public database with abundant health information on middle-aged and elderly people in the China mainland. Ultimately, our study could provide guidance to public policymakers to prevent and control hypertension and diabetes according to the local context.

## Methods

### Study population and health data

This study obtained the data from CHARLS. To ensure the adoption of the best practices and international comparability, the CHARLS was harmonized with leading international research studies following the Health and Retirement Study (HRS) model. The national baseline survey was launched between 2011 to 2012, with wave 2 in 2013, wave 3 in 2015, and wave 4 in 2018. Details on this project were presented in a previous study [22]. In brief, to ensure the national representation of the project, the study populations were selected by a four-stage, stratified, and cluster sampling method from 28 provinces and 150 counties or districts of China. We used the most recently launched data from wave 4 to calculate the prevalence of hypertension and diabetes in middle-aged and elderly people in China. In this wave, a total of 19,816 individuals completed the survey. Finally, after excluding individuals born after 1973, a total of 19,529 individuals were included in our study. Individual information on self-reported hypertension, diabetes, and smoking status was obtained from the standardized questionnaire. Information on systolic blood pressure (SBP), diastolic blood pressure (DBP), fasting plasma glucose (FPG), and hemoglobin (Hb)A1c levels were obtained from wave 3 since the information on biomarkers was not collected in wave 4.

In this analysis, the main definition of hypertension was (1) individuals who self-reported having been diagnosed with hypertension, (2) self-reported hypertension in a previous wave, or (3) an SBP of ≥ 140 mmHg, DBP of ≥ 90 mmHg, or both. Notably, since the measurements were unstable when the differences in the last two measurements were greater than 5 mmHg, another one to three measurements were taken until the differences were less than 5 mmHg. The SBP and DBP were calculated by the average of the second and third measurements [9]. Diabetes was defined as: (1) individuals who self-reported having been diagnosed with diabetes, (2) self-reported diabetes in a previous wave, (3) had a fasting plasma glucose of ≥ 7 mmol/L, or (4) an HbA1c level of ≥ 6.5%, or both [23].

As for the smoking status, individuals who self-reported having smoked in a previous wave but excluding those who answered that they never smoked in this wave, and who self-reported having smoked in this wave were defined as smokers, while the others were defined as nonsmokers.

### Air pollution data

We obtained high-spatial-resolution ground-level PM_2.5_ concentrations from the Atmospheric Composition Analysis Group at Dalhousie University [24, 25]. The data were based on the Twin MODerate Resolution Imaging Spectroradiometer (MODIS), Multiangle Imaging SpectroRadiometer (MISR), and Sea-viewing Wide Field-of-view Sensor (SeaWIFS) of the US National Aeronautics and Space Administration (NASA) inversed to obtain aerosol optical depth (AOD) data, combined with the GEOS-Chem chemical transport model, and ground monitoring data, which were incorporated into the geographically weighted regression model (GWR) to obtain ground-level annual PM_2.5_ concentration in China with a resolution of 0.01°*0.01° (approximately equal to 1 km *1 km). These data are the highest precision and largest PM_2.5_ coverage data to date.

Then, we geocoded the individuals’ addresses and assigned PM_2.5_ concentration measurements in ArcGIS software (ESRI Corporation). Specifically, the average concentration in each grid cell (0.01°*0.01°) was merged with the geographic shapefiles with information on the province boundaries of the China mainland. The PM_2.5_ exposure concentrations were then equally assigned to each province. Then we calculated the annual average PM_2.5_ from 2014 to 2018, the data in 2018 were added into primary model, other data were used in sensitivity.

### Statistical analysis

#### Descriptive analysis

The prevalence of hypertension and diabetes was calculated for 28 provinces using data from wave 4 of the CHARLS. The data relating to Ningxia, Tibet, and Hainan were determined by the average of 28 provinces. Based on the China mainland map, spatial mapping was conducted to describe the spatial distribution patterns of the prevalence of hypertension and diabetes among middle-aged and elderly people and PM_2.5_ concentrations.

#### Spatial auto-correlation analysis

Spatial auto-correlation analysis aims to describe the interrelationship of a variable among neighboring regions. This analysis can quantitatively explore the type and degree of correlation, which can provide clues for the exploration of disease risk factors from a spatial perspective. Here, Moran's index (Moran's *I*) [26] was calculated to initially understand whether there was spatial auto-correlation in the prevalence of hypertension and diabetes and PM_2.5_. The index scores range from -1.0, meaning completely spatially dispersed, to + 1.0, meaning completely spatially clustered, considering *p*-values smaller than 0.05 as statistically significant.

#### Shared Component Model (SCM)

We conducted a national cross-sectional study using the SCM to study the association of PM_2.5_ with the prevalence of hypertension and diabetes. The results were mapped to explore the spatial distribution patterns of the risk of hypertension and diabetes due to PM_2.5_. The formula was as follows [16]:

$${O}_{ji}\sim bin\left({p}_{ji},{n}_{ji}\right)$$

$$\mathrm{logit}\left({p}_{ji}\right)={\mathrm{\alpha }}_{j}+{\beta }_{j}{x}_{ji}+{eta}_{ji}$$

$${eta}_{1i}={\varphi }_{i}*\delta +{v}_{1i}$$

$${eta}_{2i}={\varphi }_{i}/\delta +{v}_{2i}$$

$${\eta }_{1}=var\left({\varphi }_{i}*\delta \right)/\left(var\left({\varphi }_{i}*\delta \right)+var\left({v}_{1i}\right)\right)$$

$${\eta }_{2}=var\left({\varphi }_{i}/\delta \right)/\left(var\left({\varphi }_{i}/\delta \right)+var\left({v}_{2i}\right)\right)$$

where *i* denoted spatial regions,* j* denoted different diseases (1 for hypertension and 2 for diabetes), *O*_*ji*_ denoted the actual number of participants with the diseases in each region, *n*_*ji*_ represented the total population surveyed in each region, ${\mathrm{\alpha }}_{j}$ was the intercept representing the baseline risk, ${x}_{ji}$ denoted the annual average PM_2.5_ exposure in each region, ${\beta }_{j}$ represented the risk of PM_2.5_ exposure for hypertension and diabetes, *p*_*ji*_ represented the potential underlying prevalence of each disease in each region, and ${\varphi }_{i}$ represented the area-level spatially shared common variability in both disease risks. The contribution of the shared component ${\varphi }_{i}$ to the risks of a specific disease was weighted by δ and 1/δ, and the logarithm of these two numbers added up to 0. ν_ji_, denoted disease-specific variability and *η*_*j*_ denoted the proportion of the shared common component's contribution to the overall spatial random effect.

To ensure that the model can be estimated, the ${\varphi }_{i}$ and *ν*_*ji*_ were further decomposed:

$${\varphi }_{i}={ush}_{i}+{ssh}_{i}$$

$${v}_{ji}={bind}_{ji}+{bspat}_{ji}$$

where *ush* and *bind* acted as surrogates for some unobserved nonspatial covariates, which were not spatially structured. Then, *ssh* and *bspat* acted as surrogates for some unobserved spatial covariates, which were spatially structured. To describe the effect of PM_2.5_, we calculated the odds ratio (OR) as exp *(β*_*j*_*)* per interquartile range (IQR) increments in PM_2.5_ concentrations.

For prior distributions, as suggested by Knorr-Held and Best [15], we assigned flat priors distributions to ${\mathrm{\alpha }}_{j}$ and ${\beta }_{j}$, and assumed that log(δ) has a normal prior distribution. To increase the identifiability and decrease the complexity of the models, conditional autoregressive (CAR) was assigned for ${\varphi }_{i}$ and ${v}_{ji}$. The Markov chain Monte Carlo simulation (MCMC) method was used to achieve Bayesian inference for the above model. To ensure that the results were reliable and easily comparable, two mutually independent Markov chains were run for the model, each with a 5000-pre-iteration burn-in period followed by 50,000 iterations. The number of pre-iterations was adjusted appropriately according to the results of the model convergence diagnosis [27].

The classical variance ratio method combined with dynamic trajectory plots and auto-correlation plots (ACF) were used for model convergence diagnosis. Meanwhile, in MCMC algorithms with multiple chains, dynamic trajectory plots were usually used to determine convergence by whether different chains had been mixed. ACF plots showing no correlation between the parameters could also suggest that the MCMC algorithm had converged. After the convergence diagnosis, the deviation information criterion (DIC) was used to select the optimal models, which could evaluate the degree of model fit to the data and the complexity of the model.

#### Sensitivity analysis

Sensitivity analysis was conducted to explore the effect of variations in PM_2.5_ in different periods and smoking status on outcome changes, and the robustness of the primary results.

Sensitivity analysis 1: The annual average PM_2.5_ concentrations in 2015, 2016, and 2017 and the average PM_2.5_ concentrations for 2016–2018 and 2014–2018 were added to the SCM, and these results in different periods were compared.

Sensitivity analysis 2: The entire study population was divided into two subgroups based on smoking status, the smoker group and the non-smoker group. The SCM was applied separately in the two subgroups, and the outcome changes were compared.

Sensitivity analysis 3: The entire population was divided into two subgroups based on age less than or equal to 60 years old and over 60 years old. The SCM was applied separately in the two subgroups and the outcome changes were compared.

Sensitivity analysis 4: The SCM was applied removing the Ningxia, Tibet and Hainan missing data, then comparing the outcome with the primary model.

Sensitivity analysis 5: We performed our models with three different priori distributions: priori 1, logdelta ~ dnorm (0.0, 10); priori 2, logdelta ~ dnorm (0.0, 30); and priori 3, logdelta ~ dnorm (0.0, 80). Then, the results were compared.

### Software

Data cleaning, preparation, descriptive analyses, and mapping were performed using R 4.1.1. Moran’s *I* was calculated in GeoDa 1.8.16.4. The SCM was run in OpenBugs 3.2.3.

## Results

### Description and spatial auto-correlation

The basic characteristics of the study individuals were summarized as follows. The study individuals consisted of 19,529 middle-aged and elderly people with ages ranging from 45 to 118, and an average age of 62.06 years. There was an approximately equal sex distribution (47.57% males and 52.43% females). Nearly 42.37% of the individuals had smoked. According to the main definition, there were 8644 patients with hypertension, for a prevalence of 44.27%, and 3600 patients with diabetes, for a prevalence of 18.44%. The average annual PM_2.5_ concentration in all provinces ranged from 4.4 μg/m^3^ to 51.3 μg/m^3^ with an average of 27.86 μg/m^3^ in 2018.

**Fig. 1:**
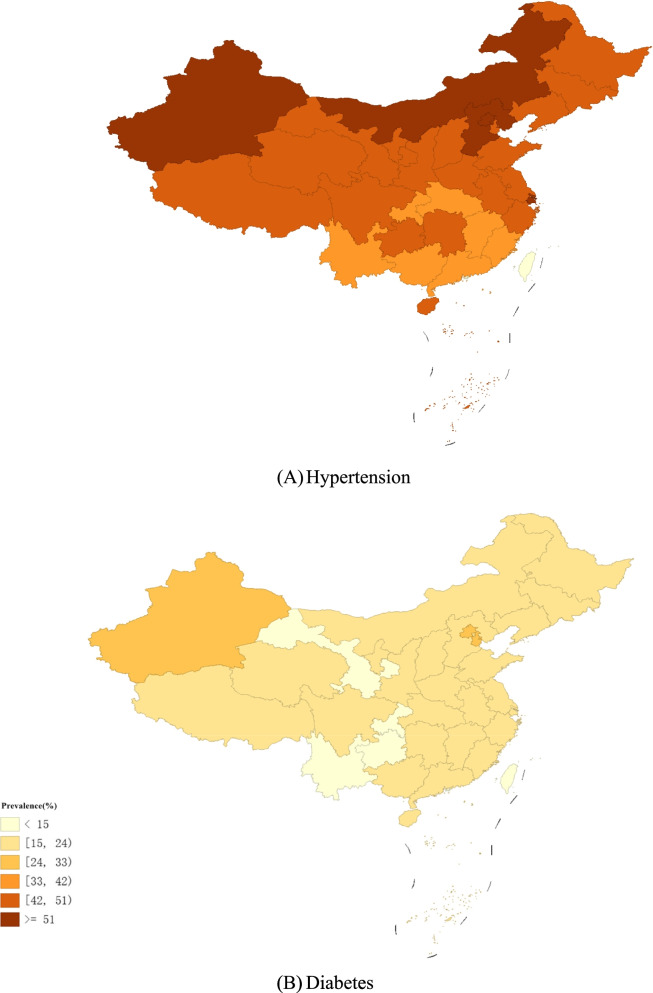
The prevalence of hypertension and diabetes among middle-aged and elderly people

The distribution of the prevalence of hypertension is shown in Fig. 1(A). The prevalence of hypertension in all provinces ranged from 34.47% to 55.88%. Xinjiang, Beijing, and Shanghai had the top three prevalence at 55.88%, 54.72%, and 54.55%, respectively. In terms of spatial distribution patterns, the prevalence of hypertension was generally higher in eastern China, especially in the northeastern regions, and lower in the central region. A higher prevalence was seen in some western provinces, such as Xinjiang.

The analysis of the prevalence of diabetes is shown in Fig. 1(B). Xinjiang, Tianjin, and Beijing had the highest prevalence at 30.39%, 25.35%, and 24.53%, respectively. The spatial distribution pattern of the prevalence of diabetes was similar to that of hypertension. We conducted spatial auto-correlation analysis of the prevalence of hypertension and diabetes. As shown in Table 1, Moran's *I* for the prevalence of hypertension and diabetes was 0.336 (*p* = 0.01) and 0.288 (*p* = 0.03), respectively, which was statistically significant. The above results indicated that there was a significant spatial auto-correlation for the prevalence of hypertension and diabetes, and spatial studies were necessary.

**Table 1:** Moran’s *I* for the prevalence of hypertension, diabetes, and PM_2.5_

	Moran’s *I*	*P*
hypertension	0.336	0.001
diabetes	0.288	0.003
PM_2.5_	0.490	0.001

**Fig. 2:**
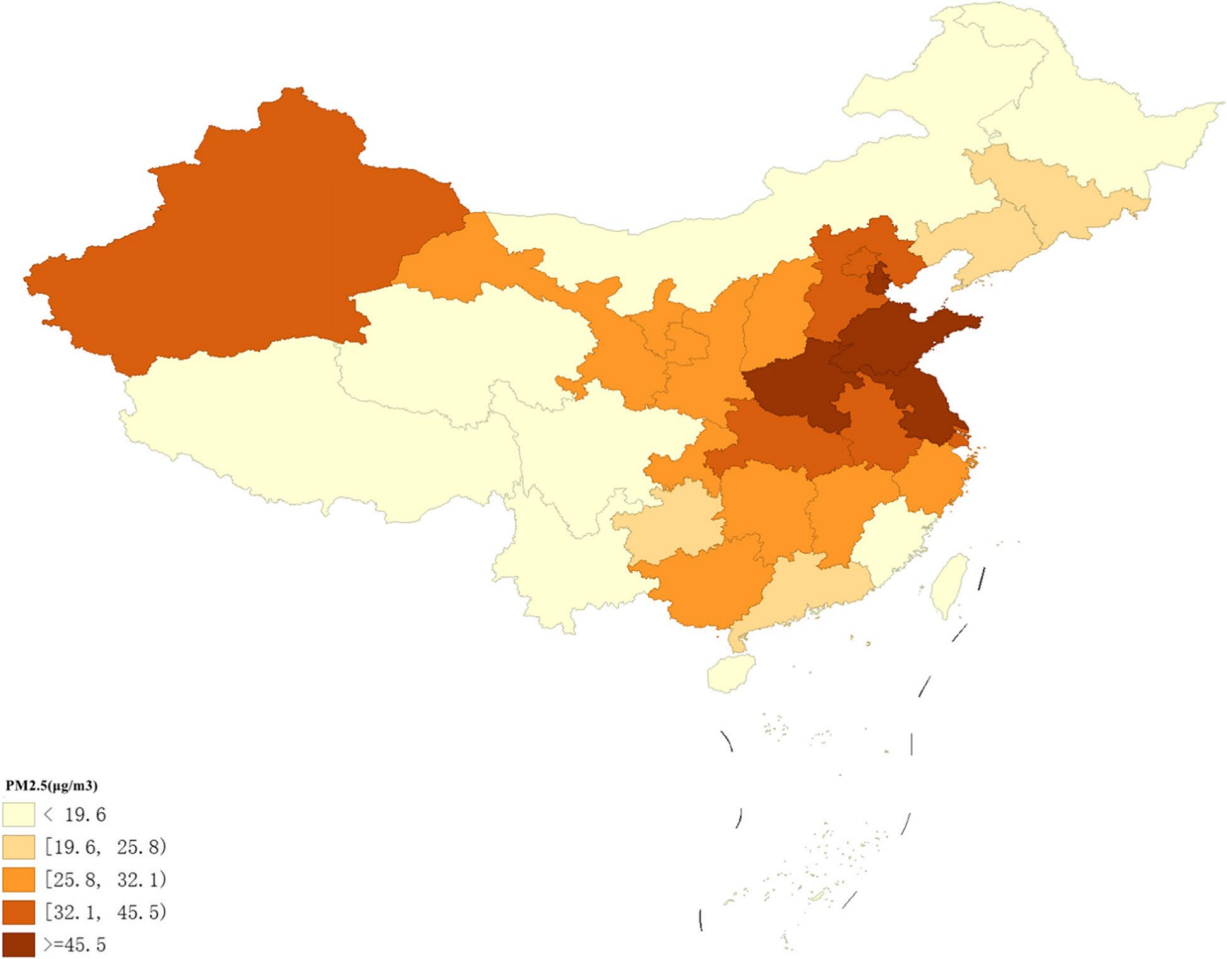
The annual average of PM_2.5_ concentration

The average annual PM_2.5_ exposure in the China mainland ranged from 4.4 μg/m^3^ to 51.3 μg/m^3^ with large regional differences among the provinces investigated. Further, spatial mapping was performed to reveal the spatial distribution patterns, and the results are shown in Fig. 2. The most serious PM2.5 problems occurred in Henan, Tianjin, and Jiangsu, with average concentrations of 51.3 ug/m3, 48.2 ug/m3, and 46.1 ug/m3, respectively. In addition to that, the average concentration of PM2.5 in Shandong and Anhui reached a relatively high level of more than 40 ug/m3. While the light pollution were reported in Tibet, Qinghai, Hainan, and Heilongjiang whose PM2.5 concentrations were 4.4 ug/m3, 12.6 ug/m3, 14.2 ug/m3, 14.2 ug/m3, respectively.Generally, the Beijing-Tianjin-Hebei region had relatively heavy pollution problems, and at most of the central regions, the PM_2.5_ concentrations were much higher than the national average. However, the air pollution problems in the northeastern and southeastern regions have been alleviated in recent years. The regions with relatively low concentrations were located in the western provinces, except for Xinjiang. The spatial auto-correlation analysis results of the average PM_2.5_ concentration are shown in Table 1. Moran's *I* was 0.490 (*p* = 0.01). The results were statistically significant, indicating that there was a significant spatial auto-correlation of PM_2.5_ concentrations and thus, spatial aggregation.

### SCM results

By applying the SCM to study the association of PM_2.5_ with hypertension and diabetes, we established model 1 without PM_2.5_ and model 2 including PM_2.5_. Both models were estimated by the MCMC method. The estimated results of the key parameters of the models are shown in Table 2. From the overall goodness-of-fit of the models, the goodness-of-fit index of model 2 (DIC = 707.5), was smaller than that of the corresponding index of model 1 (DIC = 738.7), and the pD increased slightly after adding PM_2.5_, which indicated that although model 2 was more complicated, with more parameters to be estimated than model 1, it had a more reliable model fit. Meanwhile, in model 1, *η*_1_ = 0.5619 and *η*_2_ = 0.5383 suggested that the unobserved common risk factors would determine 56.19% of the risk of hypertension and 53.83% of the risk of diabetes, respectively. From the results of model 2, the proportion of the contribution of the shared components slightly decreased to *η*_1_ = 0.5043, *η*_2_ = 0.5027 after adding PM_2.5_, suggesting that PM_2.5_ was a shared spatial risk factor for hypertension and diabetes. The posterior median of the shared component weight δ was 1.005, greater than 1, indicating that other unobserved shared risk factors affected hypertension slightly more than diabetes.

**Table 2:** SCM results

Parameters	Model 1		Model 2	
	Hypertension	Diabetes	Hypertension	Diabetes
OR.PM_2.5_			1.070(1.034,1.108)	1.149(1.100,1.200)
$\eta$	0.562(0.309,0.789)	0.538 (0.303,0.762)	0.504 (0.278,0.730)	0.503(0.279,0.726)
*δ*	1.041(0.772,1.409)		1.005(0.760, 1.324)	
DIC(pD)	738.700(2.564)		707.500(4.236)	

^*^Model 1 without PM_2.5_. Model 2 included PM_2.5_

*OR* Odds ratios, *n* the proportion of the shared common component's contribution to the overall spatial random effect, *δ* The weight of the contribution of the shared component, *DIC* Deviance information criterion

Table 2 shows that the model estimates of the OR of hypertension and diabetes per IQR increment in PM_2.5_ concentrations (16.2 μg/m^3^) were 1.070 (95% CrI: 1.034,1.708) and 1.149 (95% CrI: 1.100,1.200), respectively, both of which were statistically significant and slightly greater for diabetes than for hypertension. These results indicated that the effect of PM_2.5_ exposure on diabetes was relatively higher than that on hypertension. In terms of the risk attributed to PM_2.5_ exposure by provinces, the OR ranged from 1.019 to 1.239 for hypertension and from 1.039 to 1.553 for diabetes. The regions with the highest risk were Henan (OR for hypertension = 1.239, OR for diabetes = 1.553), Tianjin (OR for hypertension = 1.223, OR for diabetes = 1.513), and Jiangsu (OR for hypertension = 1.212, OR for diabetes = 1.486). The provinces with the lowest risks were Tibet (OR for hypertension = 1.019, OR for diabetes = 1.039), Qinghai (OR for hypertension = 1.054, OR for diabetes = 1.114), and Hainan (OR for hypertension = 1.061, OR for diabetes = 1.130).

**Fig. 3:**
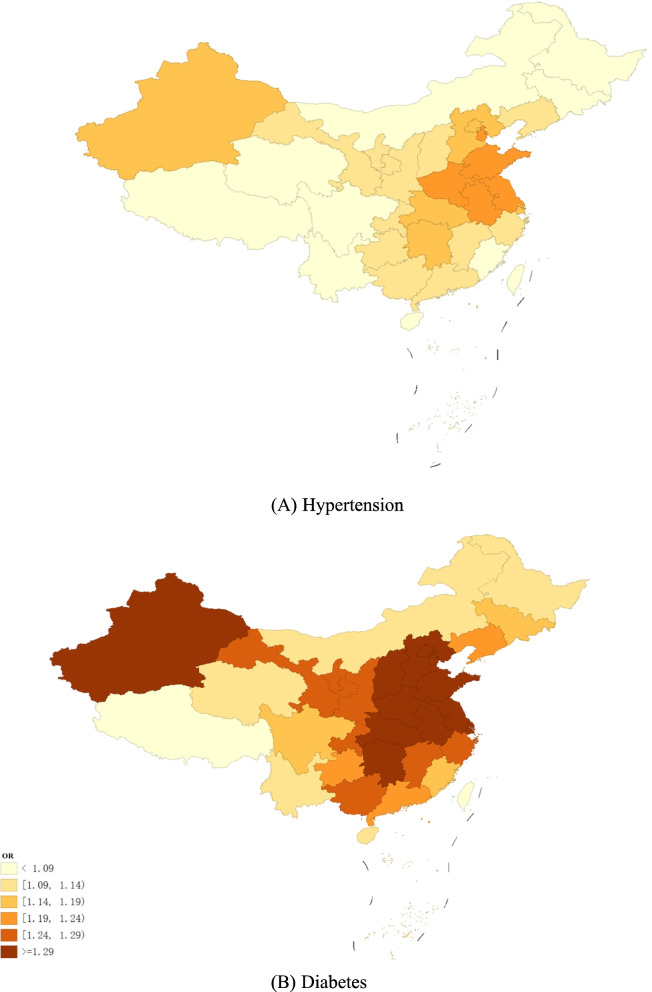
Spatial distribution of ORs of disease from PM_2.5_ exposure

From the spatial map of ORs shown in Fig. 3, the overall spatial distribution patterns were consistent with those of PM_2.5_.

### Sensitivity analyses and convergence diagnosis

**Fig. 4:**
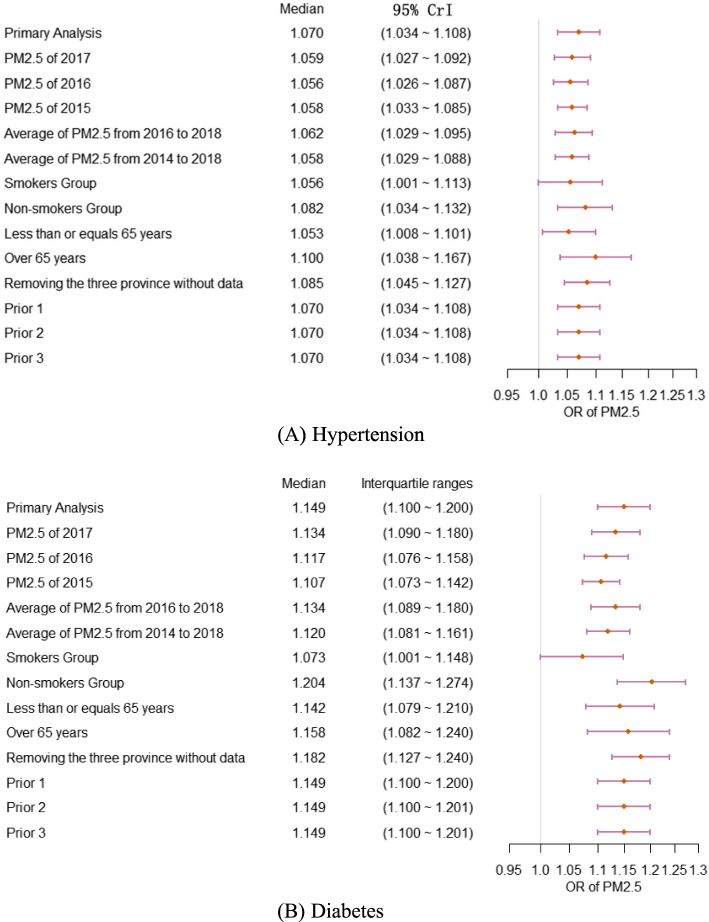
Sensitivity analysis results

Figure 4 shows the estimated results of the key parameters for all models in the sensitivity analyses. In sensitivity analysis 1, the estimated OR of PM_2.5_ for different years was not significantly different for either hypertension or diabetes. In sensitivity analysis 2, we found that the OR of PM_2.5_ for non-smokers was greater than that for smokers. To be specific, for the effect on hypertension, the OR was 1.056 (95% CrI:1.001–1.113) for smokers and 1.082 (95% CrI:1.034–1.132) for non-smokers. This difference was even more pronounced for diabetes, with an OR of 1.073 (95% CrI:1.001–1.148) for smokers and 1.204 (95% CrI:1.137–1.274) for non-smokers. These results suggested that non-smokers exposed to PM_2.5_ had a higher risk of developing both hypertension and diabetes. In sensitivity analysis 3, the OR of PM_2.5_ for the elder over 65 was slightly larger than that for less than or equal 65, and this difference was more pronounced for hypertension, with ORs were 1.053 (95% CrI:1.008 ~ 1.101) for the younger group and 1.100 (95% CrI:1.038 ~ 1.167) for the older group. For sensitivity analysis 4, the results of removing these three provinces missing data were similar to that of primary model. In sensitivity analysis 5, we applied our primary model with three different priors, and the estimated results of the key parameters were basically consistent with the primary model, indicating that the prior distribution of the primary model was set reliably and the estimated results were robust.

We also analyzed the results of convergent diagnoses. The convergence of the OR for hypertension and diabetes in the primary model is shown in Figure S1 in supplementary material. According to Brooks and Gelman statistics, all of the key variables tested fluctuated around 1, indicating that the two Markov chains showed good convergence. The model based on these prior distributions reached convergence and the estimation was relatively robust.

## Discussion

In this study, we first described the spatial distribution patterns of the prevalence of hypertension and diabetes among middle-aged and elderly Chinese people and the average PM_2.5_ concentrations in the China mainland by mapping. Moran's *I* was further calculated to confirm the existing spatial auto-correlation of the above three indicators, which were consistent with previous studies. Pei et al. reported the high prevalence of hypertension in parts of eastern China, especially the northeast, based on data from the newest China Hypertension Survey that included 451,755 participants [28]. Yin et al. analyzed the prevalence of hypertension among middle-aged and elderly people and found that there were significant spatial differences in the prevalence of hypertension in China, with a relatively higher prevalence in Shanghai, Beijing, and Inner Mongolia, and a relatively lower prevalence in Fujian [29]. As for diabetes, Zhou et al. conducted a study based on China Noncommunicable Disease Surveillance data, which included 98,058 participants, and showed that Guizhou and Yunnan had the lowest prevalence, whereas Beijing had a significantly higher prevalence, which was also consistent with the results of our study [30]. In conclusion, the effect of the differences in lifestyle, environmental factors, and socioeconomic development levels may contribute to the above geographic patterns. The PM_2.5_ results showed that the highest PM_2.5_ concentrations were in the Beijing-Tianjin-Hebei region, which was consistent with a previous study conducted in China [31]. It can be speculated that the higher PM_2.5_ concentrations in these regions were related to coal-based industries such as coal-fired power plants, and iron and steel manufacturing [32].

Furthermore, this spatial study demonstrated that long-term exposure to PM_2.5_ was significantly associated with an increased prevalence of hypertension and diabetes among middle-aged and elderly people in China. Our estimate was also robust since the PM_2.5_ concentrations of multiple years were considered in the sensitivity analyses. To our knowledge, this was the first time to study the long-term effect of air pollution on hypertension and diabetes simultaneously from spatial perspective.

Our results estimated an OR of 1.07 in the prevalence of hypertension associated with each IQR (16.2 μg/m^3^) increase in long-term PM_2.5_ exposure. Our findings were comparable to those of another study in China, which reported an OR of 1.11 with an IQR (41.7 μg/m^3^) increase in PM_2.5_ [33]. Gu et al. further confirmed the positive association based on a cohort study in China, which showed that each 10 μg/m^3^ increment in PM_2.5_ exposure increased the risk of hypertension by 11% [11]. Similarly, the American Cancer Society Cancer Prevention Study (ACSCPS) and another Canadian cohort study both showed a positive association of PM_2.5_ with hypertension [34, 35]. Our study found an OR of 1.149 for diabetes associated with an IQR (16.2 μg/m^3^) increase in long-term PM_2.5_ exposure, which was consistent with previous studies. For example, Liu et.al. found a prevalence ratio of 1.17 in type 2 diabetes mellitus (T2DM) associated with each 41.1 μg/m^3^ increase of PM_2.5_ concentration [10]. Gu et.al. estimated the percent increase in the prevalence of diabetes to be 15.66% for an increase of 10 μg/m^3^ in PM_2.5_ exposure in a study also conducted in China [12]. Several meta-analyses further confirmed the above positive associations [36–38]. Notably, the effect of PM_2.5_ exposure on hypertension and diabetes in our study was slightly less than that in previous studies. The differences may be partly explained by the differences in PM_2.5_ composition as well as the vulnerability of the populations. Moreover, compared to previous studies, which did not control for spatial confounding factors related to diseases, our study used the SCM to control for the effect of other confounding factors in the spatial perspective, making the PM_2.5_ effect estimates closer the actual effects. Although the exact biological mechanisms behind the effect of PM_2.5_ on hypertension and diabetes are unclear, several plausible explanations have been proposed. One is that the inhalation of PM_2.5_ induces the activation of pulmonary responses, which may cause an imbalance in the autonomic nervous system, resulting in high blood pressure and insulin resistance [39, 40]. Adipose tissue inflammation, oxidative stress, endothelial dysfunction, and DNA methylation can be also induced by PM_2.5_, which further results in endoplasmic reticulum stress, insulin signaling abnormalities, and apoptosis. These processes might finally result in blood pressure elevations, insulin resistance, and metabolic disturbances [41–45].

The effect of PM_2.5_ on hypertension and diabetes in this study was stronger among non-smokers, consistent with the results of Puett et al. [46] and Weinmayr et al. [47], whose studies showed that non-smokers were more sensitive to PM_2.5_ than smokers for developing hypertension and diabetes. Smoking is a risk factor for hypertension and diabetes, and the inhalation of cigarette smoke was shown to stimulate physiological responses similar to those from the inhalation of PM_2.5_, and the two related exposures shared plausible biological mechanisms, leading to adipose tissue inflammation and oxidative stress. Thus, it was plausible that smokers were much less sensitive to PM_2.5_ [48]. Another finding of sensitivity analyses was that effects of PM_2.5_ on hypertension were pronounced in the older (< 65 years) group. For older people, lifetime PM_2.5_ exposure causes longer-term oxidative stress and accumulated systemic inflammation, finally leading to older individual being more vulnerable to hypertension induced by PM_2.5_ [49]. Previous papers studying on the effects of air pollutions on hypertension also demonstrated the vulnerability of older people [50, 51].

Our study had several strengths. First, this was the first time the association of long-term exposure to PM_2.5_ with hypertension and diabetes was simultaneously explored in China, and since the two diseases have similar risk factors, they were modeled by borrowing variation information from each other, which well overcame the problem of the insufficient inclusion of control variables. Thus, the revealed associations between PM_2.5_ and hypertension and diabetes were closer to the real situations. Second, for the risk factors, PM_2.5_, with spatial correlation, we utilized Bayesian spatial analysis to explore the spatial effect, which could not be handled by classic statistical methods. Third, we combined satellite observations, chemical transport modeling, and ground-based monitoring to calculate PM_2.5_ exposure in all provinces of the China mainland, which had little bias in predicting ground-level PM_2.5_ concentrations. Fourth, this study included multiple periods of PM_2.5_ exposure concentrations in the sensitivity analysis, which ensured the robustness of the results.

Conversely, some limitations for this study should be also noted. First, the exposures were for outdoor pollution and did not estimate personal exposure levels. However, previous studies suggested a relatively strong correlation between outdoor PM_2.5_ concentrations and the corresponding individual exposure concentrations for middle-aged and elderly people [52, 53], which provided the basis for our study to use outdoor PM_2.5_ as the exposure variable. Second, this study failed to consider variations in PM_2.5_ within the province, which could lead to an underestimate of the associations. Third, this study did not consider that the use of drugs such as anti-hypertensives would be a possible effect modifier because we did not have access to drug use information in this survey. Fourth, limited evidence was available regarding the causal relationship between PM_2.5_ and hypertension and diabetes in our study. However, we considered the effect of multiple periods of PM_2.5_ exposure on the outcome, which provided a basis for a future causal study.

## Conclusions

This study found distinct spatial distribution patterns for the prevalence of hypertension and diabetes among middle-aged and elderly people and PM_2.5_ concentrations in the China mainland. Furthermore, our findings demonstrated that long-term exposure to PM_2.5_ might increase the risk of hypertension and diabetes and that non-smokers were more sensitive to the effect of PM_2.5_. Therefore, it provided a better understanding of hypertension and diabetes attributable to PM_2.5_ exposure, which will benefit policy-making and intervention designs for chronic disease prevention in China.

## Supplementary Information

**Additional file 1:** **Figure S1** (a) Convergence of key parameters for hypertension. **Figure S1** (b) Convergence of key parameters for diabetes. **Figure S1** Convergence of key parameters.

## Data Availability

The datasets generated and analyzed during the current study are available in the CHARLS website, available in http://charls.pku.edu.cn/en. And the datasets of PM_2.5_ from Atmospheric Composition Analysis Group, available in https://sites.wustl.edu/acag/datasets/surface-pm2-5/. All datasets in this study were access to public.

## Abbreviations

CHARLS: China health and retirement longitudinal study
SCM: Shared component model
IQR: Interquartile range
OR: Odds ratio
CrI: Credible interval
BHM: Bayesian hierarchical model
GBD: The global burden of disease study
COPD: Chronic obstructive pulmonary disease
IHD: Ischemic heart disease
MCMC: Markov chain monte carlo
HRS: The health and retirement study model
SBP: Systolic blood pressure
DBP: Diastolic blood pressure
MODIS: The Twin MODerate resolution imaging spectroradiometer
MISR: Multiangle imaging spectroradiometer
SeaWIFS: Sea-viewing wide field-of-view sensor
NASA: US National Aeronautics and Space Administration
AOD: Aerosol optical depth data
GWR: Geographically weighted regression model
Moran’s *I*: Moran's index
CAR: Conditional autoregressive
ACF: Auto-correlation plots
DIC: Deviation information criterion
