# Supplementary material for: Association of long-term exposure to PM2.5 with hypertension and diabetes among the middle-aged and elderly people in Chinese mainland: a spatial study
Source: BMC Public Health. 2022 Mar 22;22:569. doi: 10.1186/s12889-022-12984-6 (PMC8941772; doi:10.1186/s12889-022-12984-6)
Supplement: Supplementary file 1 — Additional file 1: Figure S1 (a) Convergence of key parameters for hypertension. Figure S1 (b) Convergence of key parameters for diabetes. Figure S1 Convergence of key parameters. [file 12889_2022_12984_MOESM1_ESM.docx]

**supplementary material**

**Figure S1 (a)** Convergence of key parameters for hypertension

**Figure S1 (b)** Convergence of key parameters for diabetes

**Figure S1** Convergence of key parameters
